# Prenatal diagnosis identifies compound heterozygous variants in *RYR1* that causes ultrasound abnormalities in a fetus

**DOI:** 10.1186/s12920-022-01358-x

**Published:** 2022-09-21

**Authors:** Qiuling Zhao, Xiaoduo Li, Li Liu, Xu Zhang, Xin Pan, Hong Yao, Yongyi Ma, Bo Tan

**Affiliations:** 1 Department of Gynecology and Obstetrics, The Second Affiliated Hospital of Chongqing Medical University, Chongqing, China; 2 Department of Gynecology and Obstetrics, Southwest Hospital, Third Military Medical University (Army Medical University), Chongqing, China; 3 Qijiang Maternal and Child Health Hospital, Chongqing, China; 4 Institute of Pathology, Southwest Hospital, Third Military Medical University (Army Medical University), Chongqing, China

**Keywords:** Ryanodine receptor type 1 gene, *RYR1*-related disorders, Ultrasound abnormalities, Splice-site variant, Whole-exome sequencing

## Abstract

**Objective:**

We presented a non-consanguineous healthy Chinese couple with five pregnancies, three early miscarriages, the fetus II-2 and II-5 with similar abnormal phenotypes of fetal hydrops, scoliosis, fetal akinesia and polyhydramnios. This study aimed to uncover the molecular etiology of this family with a history of multiple adverse pregnancies.

**Materials and methods:**

DNA extracted from the fifth fetal umbilical cord and parents’ peripheral blood were subjected to SNP-array and whole exome sequencing. The result was verified by Sanger sequencing. Functional characterization of the c.2682G > C (p.Ile860_Pro894del) variant was completed by minigene splicing assay.

**Results:**

Trio whole-exome sequencing has identified compound heterozygous variants in *RYR1* (c.2682G > C; p.Ile860_Pro894del and c.12572G > A; p.Arg4191His) in fetus II-5. The variant c.2682G > C (p.Ile860_Pro894del) comes from the father and the c.12572G > A (p.Arg4191His) comes from the mother. The c.2682G > C (p.Ile860_Pro894del) affects the splice site resulting in exon 21 skipping, therefore is classified as likely pathogenic. The c.12572G > A (p.Arg4191His) locates in the C-terminal hot spots region of the *RYR1*, classified as of uncertain significance.

**Conclusions:**

We report the first prenatal case of *RYR1*-related disorders in Chinese population, expanding the variant spectrum of *RYR1* in fetuses.

**Supplementary Information:**

The online version contains supplementary material available at 10.1186/s12920-022-01358-x.

## Introduction

The skeletal muscle ryanodine receptor (*RyR1*) locates in the sarcoplasmic reticulum (SR) of skeletal muscle and releases calcium (Ca^2+^) into the cytoplasm upon depolarization of the sarcolemma, playing a significant role in excitation–contraction coupling [1]. As one of the largest genes in the human genome, the *RYR1* gene includes 106 exons and covers 15.4 kb on chromosome 19q13.1 [2].

Variant in the *RYR1* gene is first described in a Canadian family with malignant hyperthermia (MH) [3]. *RYR1* variants have subsequently been identified in various myopathies including central core disease (CCD), multi-minicore disease (MmD), centronuclear myopathy (CNM), congenital fiber-type disproportion (CFTD), core-rod myopathy (CRM), King-Denborough syndrome, dusty core disease (DuCD), samaritan myopathy, a late-onset axial myopathy, fetal akinesia and lethal multiple pterygium syndrome (LMPS) [4–9]. The age of onset, clinical presentation, disease progression and severity of these myopathies vary widely. With the constant advancement of next generation sequencing, the *RYR1* disease spectrum has further expanded. Clinical and pathological overlaps make the differential diagnosis of *RYR1*-related myopathies difficult. Lawal et al. propose *RYR1*-related disorders (*RYR1*-RD) as a unified nomenclature to describe this complex and evolving disease spectrum [10].

In this study, we report a Chinese couple with five pregnancies who had three early miscarriages and two fetal losses because of a primary myopathy characterized by fetal akinesia, polyhydramnios, umbilical cord cyst, abnormality of the vertebral column, abnormality of the scrotum, rocker bottom foot, absence of stomach bubble and hydrops fetalis. However, no further genetic tests were carried out on the first four fetuses as their samples were not available. Abnormal karyotype and pathogenic CNV of the fifth fetus and parents had been excluded. Trio whole-exome sequencing was performed to identify the genetic causes of these symptoms. Lethality of the condition and potential phenotype recurrence based on the reproductive history suggested an autosomal recessive condition.

## Material and methods

### Clinical report

**Fig. 1:**
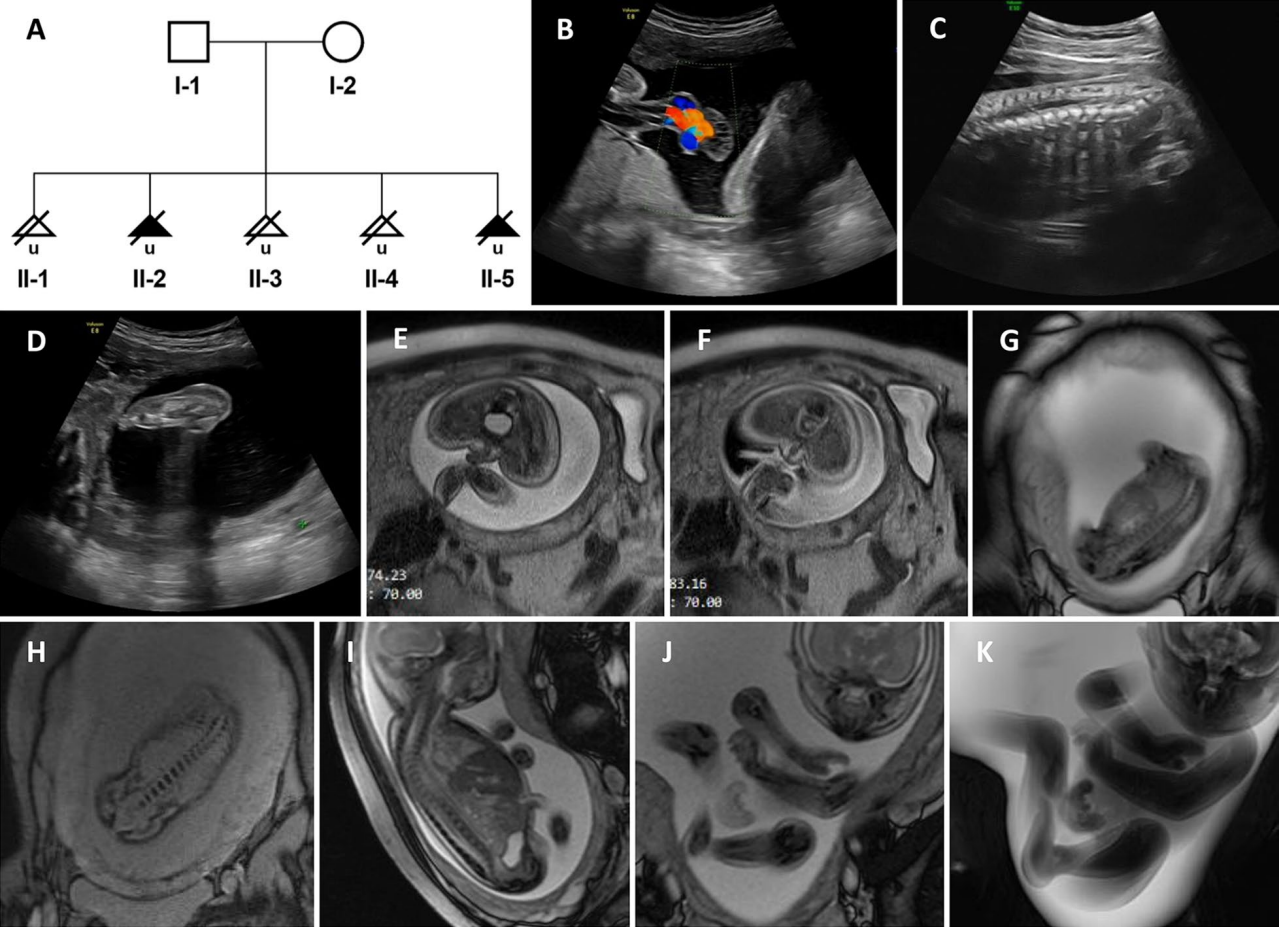
**A** Pedigree of the non-consanguineous Chinese family. **B**–**D** Fetal II-5 ultrasound findings at 25 weeks of gestation showing the umbilical cord cysts. (**B**), abnormal conus medullaris morphology (**C**), and thickened heel subcutaneous tissue (**D**). **E**–**K** Fetal II-5 magnetic resonance imaging showing abnormality of the scrotum (**E**–**F**), thoracic scoliosis (**G**–**H**), abnormal conus medullaris morphology (**I**), hand clenching (**J**), and rocker bottom foot (**K**)

We report a non-consanguineous Chinese family that had five pregnancies affected by *RYR1*-related disorders (Fig. 1A). The mother was a 27 year-old woman. The couple was healthy and had no personal or family history of the neuromuscular disorder or malignant hyperthermia. The first pregnancy (II-1) ended in abortion.

The second pregnancy (II-2) was complicated by fetal ultrasound findings that polyhydramnios, single umbilical artery, intrauterine growth retardation, and absence of stomach bubble at 28 weeks of gestation. At post mortem examination there was spina bifida and scoliosis associated with vertebral segmentation defects. An additional genetic analysis was not selected.

Their third and fourth pregnancy (II-3 and II-4) were terminated, because of no yolk sac in early pregnancy.

Subsequently, fetal ultrasound of the fifth pregnancy (II-5) revealed increased nuchal translucency (NT: 8.8 mm), hand clenching, adducted thumb, absence of stomach bubble on fetal sonography, polyhydramnios, umbilical cord cysts, abnormal conus medullaris morphology, and thickened heel subcutaneous tissue (Fig. 1B–D) at 25 weeks’ gestation. Magnetic resonance imaging (MRI) showed abnormality of the scrotum, thoracic scoliosis, abnormal conus medullaris morphology, hand clenching, rocker bottom foot, fetal immobility, and subcutaneous oedema (Fig. 1E–K). The fetal and the couple's karyotypes were normal. Based on the multiple malformation outcomes, the pregnancy was terminated 3 weeks later. Whole exome sequencing analysis was performed using the fetal umbilical cord and parents’ blood. Unfortunately, the II-5 muscle samples were not retained for histological studies.

All participants provided written informed consent. The study protocols were approved by the ethics committee from the Second Affiliated Hospital of Chongqing Medical University.

### SNP-array and exome sequencing

Genomic DNA was extracted from the umbilical cord of II-5 and peripheral blood of parents, using DNeasy Blood & Tissue kit (Qiagen, Hilden, Germany). Single nucleotide polymorphism array analysis was performed on the Affymetrix CytoScan platform (Affymetrix, Santa Clara, CA, USA) following the protocol. Exome capture was performed on 3 µg of genomic DNA sample using the Agilent SureSelectXT V5 capture kit (Agilent Technologies, Santa Clara, CA), and then sequenced on Illumina HiSeq 2000 (Illumina, San Diego, CA) with 100-bp paired-end reads.

### Variant annotation and selection

SNP-array data were analyzed by Chromosome Analysis Suite 3.2 (Affymetrix, Santa Clara, CA, USA). Raw data were mapped to the human reference genome (GRCh37/hg19) using Burrows‐Wheeler Aligner [11] and variants calling using Genomic Analysis Tool Kit best practices [12]. Variants were annotated by ANNOVAR using ExAC, 1000 Genomes, Genome Aggregation Database (gnomAD) and other public databases [13]. Stepwise filtering included the removal of variants with quality scores < 20 and allele counts < 10X, minor allele frequency (MAF) > 1%, common SNPs, intergenic and 3’/5’ UTR variants, non-splice-related intronic variants, benign variants and likely benign variants, and synonymous variants. Only variants matched the inheritance model of the family and fetuses’ phenotypes were kept. The pathogenicity of variants was interpreted and classified according to the American College of Medical Genetics and Genomics (ACMG) and the Association for Molecular Pathology (AMP) [14].

### Sanger sequencing

Parental blood samples as well as amniotic fluid of the fetus II-5 were used for sanger sequencing after WES analysis. Primers were designed using Primer3 online (http://primer3.ut.ee/). Primers RYR1_1 (Forward, 5’-AGAACATCCACGAGCTCTGG-3’ and Reverse, 5’-CCAGACTATGACCCCTGACC-3’) for variant c.2682G > C (p.Ile860_Pro894del) and RYR1_2 (Forward, 5’-ATCCTTGAGTACTTCCGCCC-3’ and Reverse, 5’-TGAGGGTGCAGGAAGTGAG-3’) for variant c.12572G > A (p.Arg4191His) were used to span the sequences. Sequencing was performed in both directions by BGI (Chongqing, China). The reference sequence of *RYR1* used was GenBank NM_000540.3.

### In silico analysis

In silico prediction tools (SIFT, PolyPhen-2, Mutation Taster, and LRT) were used to assess variant pathogenicity and potential functional effects. Online analysis tools (Human Splicing Finder, and NetGene2) were used to evaluated potential effects on splicing. NCBI Protein blast was used to analyze the conservation of amino acid residue alteration by comparing across different species.

### Minigene splicing assay

We performed functional analysis of the c.2682G > C (p.Ile860_Pro894del) variant with an in vitro minigene assay. Genomic DNA of the parents was amplified using specific primers (RYR1_Ex20_F: 5′-CCCAAGCTTCCGCCATGGTGAATTCAAGT-3′, RYR1_Ex22_R: 5′-ATAGTTTAGCGGCCGCCTCTGGAAGGCTGTGGAAGT-3′) for exons 20, 21, 22 and intron sequences (3432 bp). The amplified minigene products were cloned into the pcDNA3.1(+) plasmid (Invitrogen) at the Hind III and Not I sites. After verifying by sequencing, we obtained a homozygous mutant-type hybrid minigene from the father (pcDNA3.1-MT) and a wild-type hybrid minigene from the mother (pcDNA3.1-WT). Then, hybrid minigenes were transfected into HEK293T cells transiently with the Lipofectamine 2000 reagent (Invitrogen). After 48 h, RNA was extracted from each plate of cells and RT‐PCR was performed with the same primers. RT‐PCR products were analyzed by electrophoresis analysis and Sanger sequencing.

## Results

**Fig. 2:**
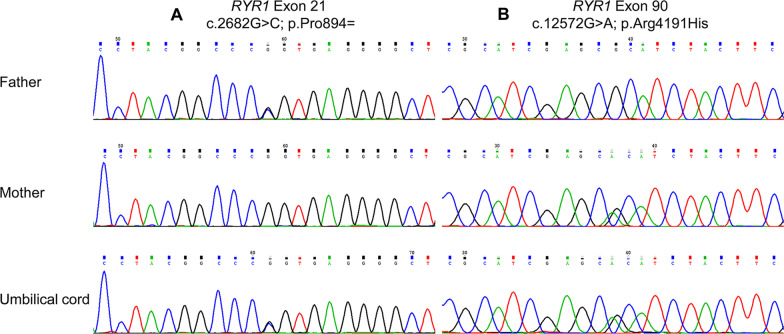
Sanger sequencing validation of *RYR1* variants identified by exome sequencing. **A** Variant c. 2682G > C in the *RYR1* gene in heterozygous state (father). **B** Variant c. 12572G > A in the *RYR1* gene in heterozygous state (mother)

G-banding analysis showed that the karyotype of the fetus II-5 and the couple were normal. No CNVs were identified by SNP-array in fetus II-5 and parents. Trio WES analysis found compound heterozygous variants in *RYR1*, a c.2682G > C (p.Ile860_Pro894del) variant that was inherited from the father and a c.12572G > A (p.Arg4191His) variant that was inherited from the mother. Subsequently, sanger sequencing revealed the same results (Fig. 2).

The c.2682G > C (p.Ile860_Pro894del) variant is novel (i.e., not reported in ExAC/gnomAD, ClinVar, 1000 Genomes, or HGMD databases and not published to date). This variant is located at the last nucleotide of exon 21 and is predicted to cause *RYR1* alternative splicing by Mutation Taster, NetGene2 and HSF 3.0. To characterize the abnormal splicing, minigene Splicing Assay was performed.

**Fig. 3:**
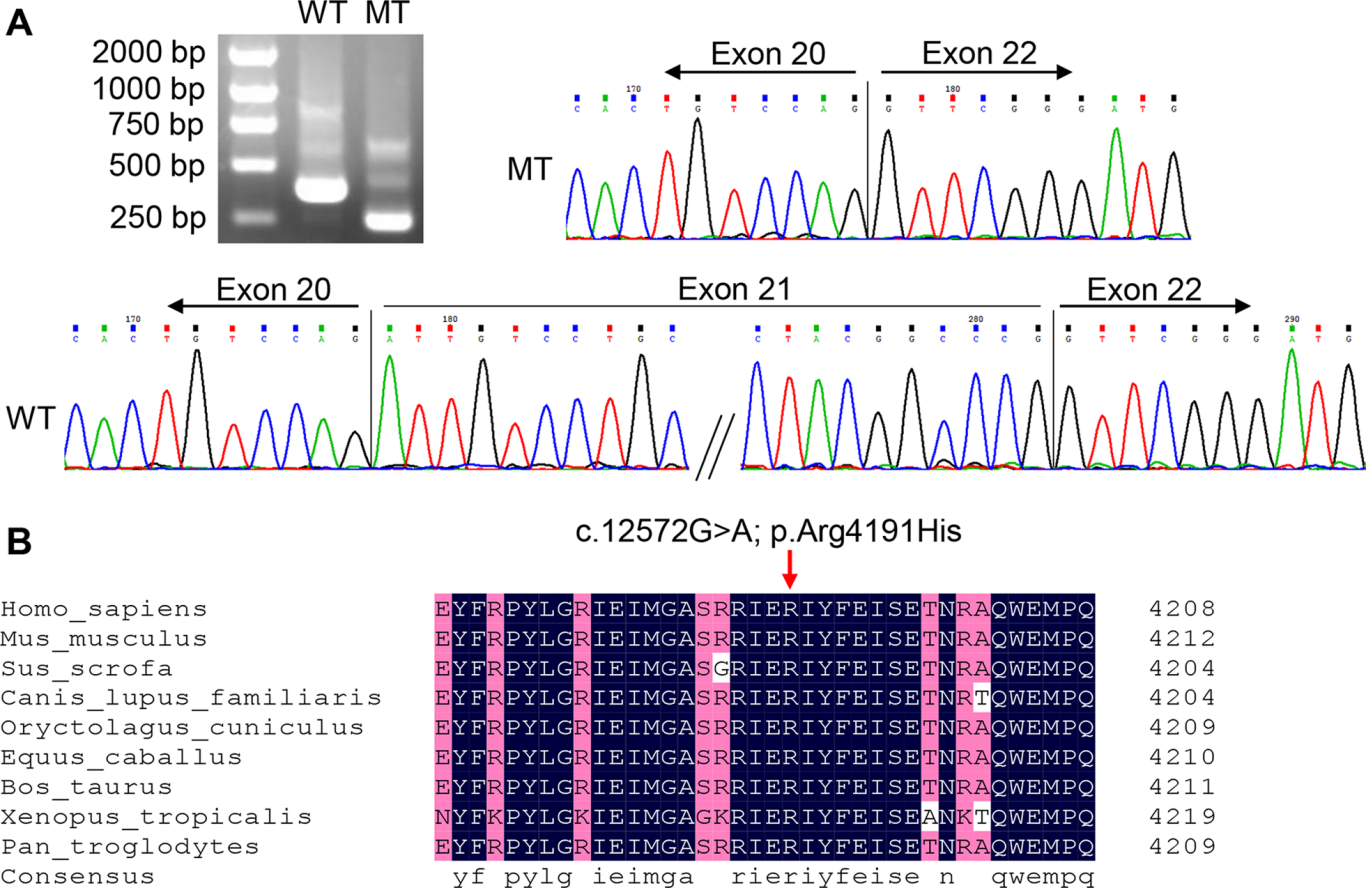
**A** Minigene splicing assay. The WT higher band represented correctly spliced exons, whereas the MT lower band represented the *RYR1* deletion 105 bp in exon 21. Sequencing revealed the MT-type was missing the entire exon 21 sequence due to the c.2682G > C variant. **B** Conservation analysis. The residue Arg4191 was highly conserved among nine different species

Splicing products of c.2682G > C (p.Ile860_Pro894del) was analyzed by RT-PCR. Agarose gel electrophoresis showed that pcDNA3.1-WT plasmid produced a 361 bp band, while pcDNA3.1-MT generated a 256 bp band (Fig. 3A and Additional file 1). Sequencing analysis confirmed that the product of pcDNA3.1-WT was consistent with the reference sequence. In contrast, sequencing pcDNA3.1-MT indicated a complete skipping of exon 21 (Fig. 3A). That is, the c.2682 G > C (p.Ile860_Pro894del) variant caused a deletion of 105 bp in exon 21. According to the AMP/ACMG guidelines for the interpretation of sequence variants, the novel c.2682 G > C (p.Ile860_Pro894del) variant was assessed to be likely pathogenic.

The population frequency of this c.12572G > A (p.Arg4191His) variant is extremely low (MAF of 0.000008). The variant was previously detected (rs765730833), but had not been reported in individuals with *RYR1*-related disease. It was highly conserved and was predicted deleterious by SIFT, Polyphen‐2, and LRT. The c.12572G > A (p.Arg4191His) variant located in the C-terminal domain on exon 90, was highly conserved across species (Fig. 3B). According to the ACMG/AMP standards and guidelines, the variant is uncertain significance.

## Discussion

With advances in genetic analysis techniques, the spectrum of *RYR1*-RD has gradually expanded. *RYR1* has been repeatedly reported to be associated with severe congenital onset in the neonatal period [9, 15–20]. A severe form of central core disease (CCD) had been reported in seven cases with fetal akinesia, multiple arthrogryposis and hydramnion during pregnancy. Ultimately, two fetuses died before birth and three infants died before their first birthday [15]. In 2016, Lethal multiple pterygium syndrome (LMPS) was considered at the extreme end of the *RYR1*-RD spectrum. It usually presents as prenatal growth failure with pterygium present in multiple areas, severe arthrogryposis, akinesia and fetal hydrops [19]. In this report, we describe two fetuses with fetal akinesia, polyhydramnios, multi-malformation and hydrops fetalis presented with some features of CCD and LMPS. Since there is no typical multiple pterygium phenotype and no fetal tissue remains for histopathological studies, we can’t identify the disease subtype. Based on fetuses’ phenotype and WES test results, we use *RYR1*-RD to describe the disease we found.

In these cases, there are both dominant inheritance and recessive inheritance. Dominant variants are found to concentrate at three “hot spots” [21]: N-terminal (residues p.Met1-p.Arg614), central (p.Arg2163–p.Arg2458) and C-terminal (p.Arg4136-p.Pro4973). While recessive variants are distributed through the entire *RYR1* gene. Dominant variants are clinically characterized by mild hypotonia and non-progressive weakness, while recessive variants may cause early-onset and severe myopathy. And, patients with at least one hypomorphic allele (nonsense, frameshift, indel, or splice) had increased disease severity [22, 23]. To date, the molecular disease patho-mechanisms of *RYR1*-RD caused by dominant versus recessive variants are not completely clear.

Here, we reported compound heterozygous variants (splicing and missense) in *RYR1* in a fetus. It was consistent with recessive inheritance pattern in this family and leads to more severe phenotype. The variant (c.2682G > C) was the skipping of the entire exon and consequently the loss of the c.2578_2682 in the mRNA, resulting in an in-frame deletion (p.Ile860_Pro894del). The largest RyR1 domain was the myoplasmic domain, also known as the RyR1 foot region, which consisted of the first 3613 amino acid residues [24]. The myoplasmic domain formed key inter-subunit interactions and housed binding sites for channel activity modulator proteins calmodulin, S100A1, FK506-binding protein (FKBP12), and dihydropyrine receptor [25–27]. Another variant c.2682G > A (p.Pro894 =) at the same site had been reported in an individual affected with multiminicore disease. Furthermore, previously missense substitutions within this deletion (c.2654G > T; p.Arg885Leu and c.2603G > A; p.Arg868His) had been reported in dominantly inherited central core myopathy [28]. Thirty five amino acid deletions may result in an alteration of the protein’s secondary structure, which then may affect protein interactions. So, the novel splicing variant was classified as “likely pathogenic”.

The second missense variant (c.12572G > A) located in C-terminal ‘hot spots’ had been previously reported. The variant c.12572G > A resulted in an aromatic amino acid (His) was substituted for an aliphatic amino acid (Arg) at amino acid position 4191. The maternal p.Arg4191His variant affected a highly conserved amino acid located in the M2–M3 cytoplasmic loop of the channel release domain according to the RYR1 10-transmembrane domain-based model [29]. In addition, the variation was located in a previously predicted EF-hand pair that constituted the conserved Ca^2+^-binding domain in RyRs [30]. However, the p.Arg4191His had also been reported to be located in the thumb-and-forefinger domain of RyR1, which connects to the C-terminal extension of the S6 helix. The TaF domain is an ATP ligand binding site and close to putative Ca^2+^ binding site. It is part of the activation module for conformational changes upon activation and opening of RyR1 [24, 31]. Furthermore, the central domain contained amino acids 3668–4251 and constituted a docking station for a variety of allosteric regulatory factors including proteins and small molecules [32]. Therefore, the p.Arg4191His directly or indirectly altered Ca^2+^ sensing or the interaction with RyR1 agonists or antagonists, thereby interfering with channel activity. According to the ACMG and AMP criteria, this variant was classified as “uncertain significance”.

Taken together, we describe a ‘severe neonatal form’ of *RYR1*-RD with the antenatal onset of symptoms, two fetuses in a non-consanguineous healthy Chinese family presented with an abnormality of the vertebral column, absence of stomach bubble, polyhydramnios, and multi-malformation during pregnancy. They were terminated at 28 and 25 weeks of gestational age, respectively. Trio-WES to identified missense variant and splicing variant in *RYR1* and we confirmed the pathogenicity of the splicing variant using a simple in vitro reporter minigene assay. In our case, clinical diagnosis was difficult as we lacked fetal muscle biopsy specimens to confirm the disease and the parental biopsies were not available for morphological investigation. Further genetic tests could not be carried out on the other four fetuses in this family, as no samples were available. We can only infer the same molecular etiology by phenotypic recurrence. This is the limitation of this study. We advocate that future fetuses with similar clinical presentation are examined with muscle biopsy of affected muscle. Moreover, genome-wide sequencing, like WES, would be a helpful and effective testing in prenatal diagnosis, especially for those fetuses without definite clinical differential diagnosis. Although some *RYR1*-RD cases of the severe neonatal phenotype had been reported abroad, this is the first report of variants in the *RYR1* gene involved in a severe form of *RYR1*-RD in China. We reveal new findings expanding the disease spectrum of *RYR1* gene variants.

## Supplementary Information

**Additional file 1.** The original image of figure 3A.

## Data Availability

The clinical case records of our patients are available from the corresponding author on reasonable request. The whole exome sequencing data generated during the current study have been deposited in the Genome Sequence Archive (GSA). The direct web links to this dataset is https://ngdc.cncb.ac.cn/gsa-human/s/1YQ2331a.
