# Supplementary figures and images for: Prenatal diagnosis identifies compound heterozygous variants in RYR1 that causes ultrasound abnormalities in a fetus
Source: BMC Med Genomics. 2022 Sep 21;15:202. doi: 10.1186/s12920-022-01358-x (PMC9490926; doi:10.1186/s12920-022-01358-x)

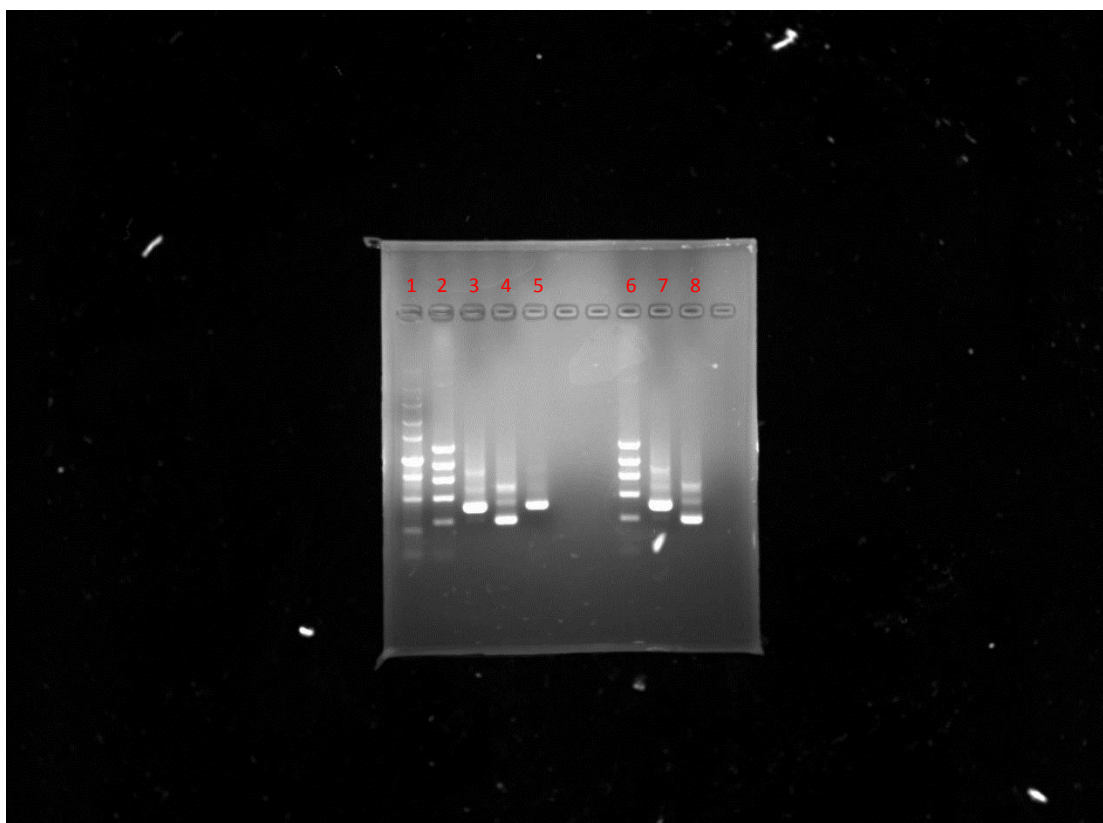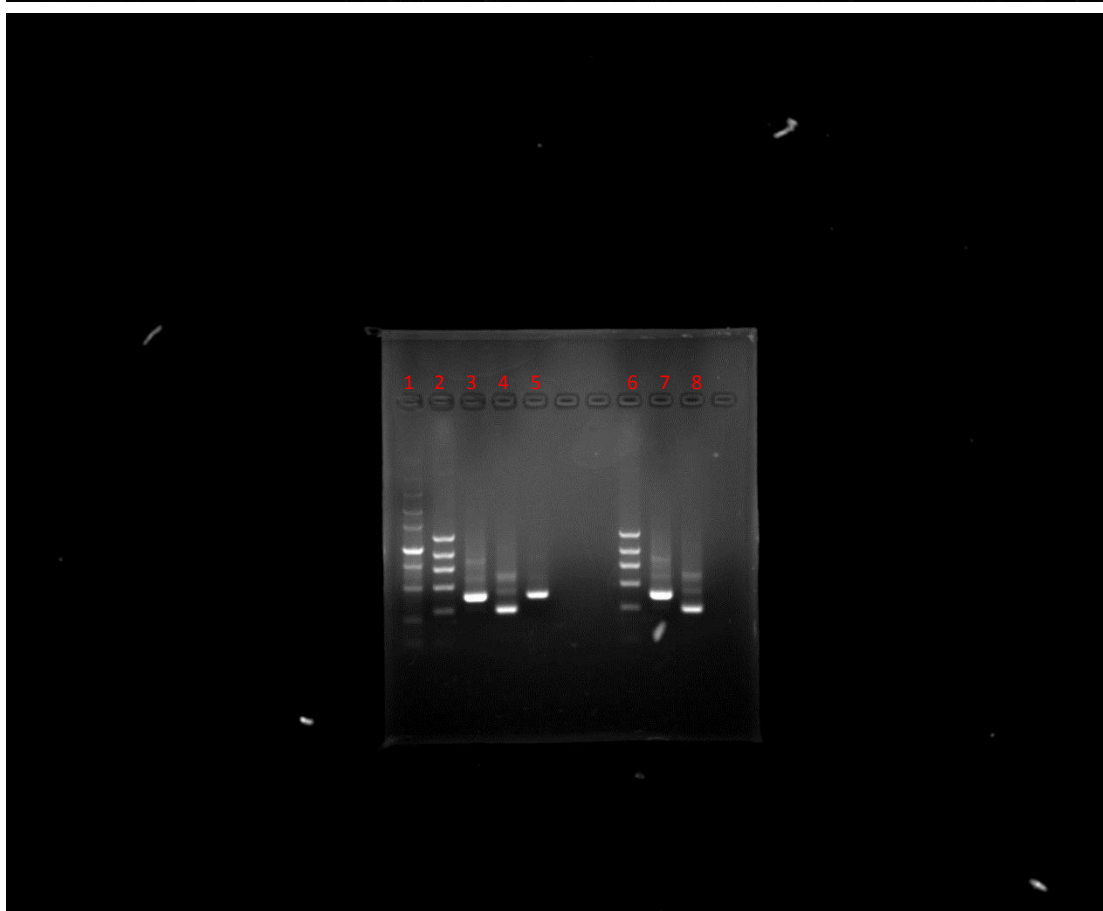

1 DL10000 DNA Marker

2 DL2000 DNA Marker  
3 pcDNA3.1-WT  
4 pcDNA3.1-MT  
5 pcDNA3.1-WT  
6 DL2000 DNA Marker  
7 pcDNA3.1-WT  
8 pcDNA3.1-MT

Supplement: Supplementary file 1 — Additional file 1. The original image of figure 3A. [file 12920_2022_1358_MOESM1_ESM.pdf]
